# Supplementary material for: The interactome of intact mitochondria by cross-linking mass spectrometry provides evidence for coexisting respiratory supercomplexes
Source: Mol Cell Proteomics. 2017 Dec 8;17(2):216–32. doi: 10.1074/mcp.RA117.000470 (PMC5795388; doi:10.1074/mcp.RA117.000470)
Supplement: Supplemental Data [file supp_RA117.000470_133922_0_supp_23978_fzffwf.zip › spectra_annotation/mito_spectra_annotation/322-1-10-1-10-1.pdf]

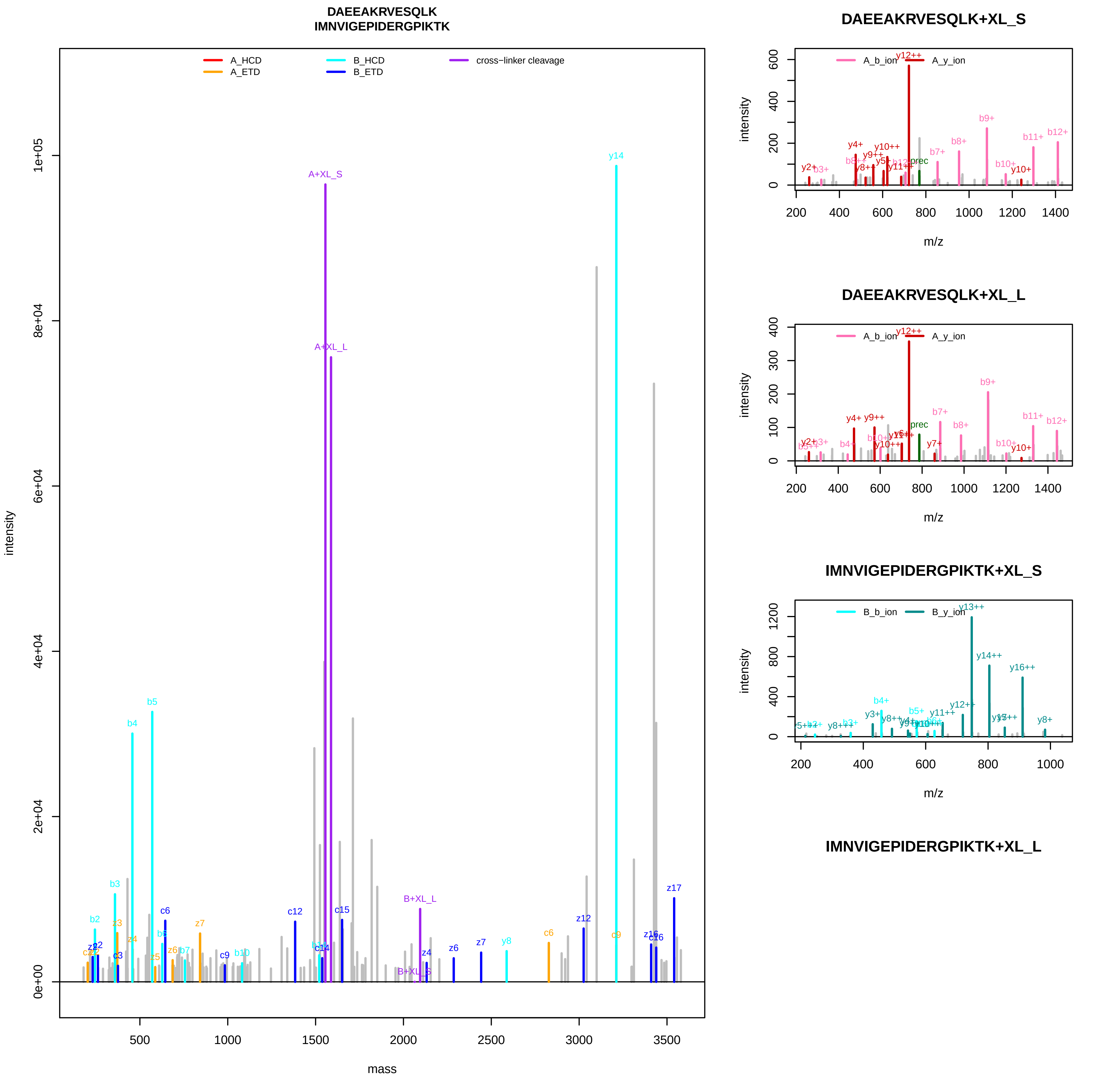
DAEEAKRVESQLK
IMNVIGEPIDERGPIKTK
A_HCD
A_ETD
B_HCD
B_ETD
cross−linker cleavage
intensity
mass
0e+00
2e+04
4e+04
6e+04
8e+04
1e+05
500
1000
1500
2000
2500
3000
3500
y14
A+XL_S
A+XL_L
B+XL_L
B+XL_S
b2
b3
b4
b5
c6
z7
c9
b10
c12
c14
c15
z4
z6
z7
y8
c6
z12
c9
z16
c16
z17
DAEEAKRVESQLK+XL_S
DAEEAKRVESQLK+XL_L
IMNVIGEPIDERGPIKTK+XL_S
IMNVIGEPIDERGPIKTK+XL_L
A_b_ion
A_y_ion
B_b_ion
B_y_ion
m/z
y12++
prec
b9+
b8+
b7+
b11+
b12+
b10+
y10+
y4+
y10++
y9++
y2+
y13++
y14++
y16++
y12++
y11++
b4+
b5+
y3+
y8+
